# Pathogenesis and genetic characteristics of novel reassortant low-pathogenic avian influenza H7 viruses isolated from migratory birds in the Republic of Korea in the winter of 2016–2017

**DOI:** 10.1038/s41426-018-0181-3

**Published:** 2018-11-15

**Authors:** Yu-Na Lee, Sun-Ha Cheon, Eun-Kyoung Lee, Gyeong-Beom Heo, You-Chan Bae, Seong-Joon Joh, Myoung-Heon Lee, Youn-Jeong Lee

**Affiliations:** 1 Avian Influenza Research & Diagnostic Division, Animal and Plant Quarantine Agency, 177 Hyeoksin 8-ro, Gimcheon-si, Gyeongsangbuk-do, 39660 Republic of Korea; 2 Avian Disease Division, Animal and Plant Quarantine Agency, 177 Hyeoksin 8-ro, Gimcheon-si, Gyeongsangbuk-do, 39660 Republic of Korea

## Abstract

In this study, we characterized H7 subtype low-pathogenicity (LP) influenza A viruses (IAVs) isolated from wild bird habitats in the Republic of Korea from 2010 to early 2017. Through national surveillance, 104 H7 IAVs were isolated, accounting for an average of 14.9% of annual IAV isolations. In early 2017, H7 subtypes accounted for an unusually high prevalence (43.6%) of IAV detections in wild birds. Phylogenetic analysis revealed that all the viruses isolated in the winter of 2016–2017 fell within cluster II of group C, belonging to the Eurasian lineage of H7 IAVs. Notably, cluster II of group C included the H7 gene from the highly pathogenic H7N7 IAV that was detected in northeastern Italy in April of 2016. Through a gene-constellation analysis, the H7 LPIAVs that we isolated constituted ≥11 distinct genotypes. Because the viruses belonging to the genotypes G2.1 and G1 were observed most frequently, we compared the replication and transmission of representative viruses to these genotypes in specific-pathogen-free chickens. Notably, the representative G2.1 strain was capable of systemic replication and efficient transmission in chickens (as evidenced by virus isolation and histopathological examination) without any clinical signs except mortality (in one infected chicken). The efficient subclinical viral replication and shedding of the G2.1 virus in chickens may facilitate its silent spread among poultry after introduction. Given that wild birds harbor novel strains that could affect poultry, our results highlight the need for enhanced IAV surveillance in both wild birds and poultry in Eurasia.

## Introduction

*Influenza A virus* (IAV) is an enveloped virus with a segmented, single-stranded, negative-sense RNA genome that is the type species of the *Influenza A virus* genus in the family *Orthomyxoviridae*^1^. *Influenza A virus* is classified into serotypes according to the presence of hemagglutinin (HA) and neuraminidase (NA) protein subtypes. Currently, 16 HA and nine NA subtypes are known among circulating IAVs in the aquatic-bird reservoir, and the H17N10 and H18N11 strains have been identified in bats^2^. IAVs evolve at a rapid rate which primarily occurs by two mechanisms, point mutation, and reassortment^3^. The lack of a polymerase proofreading mechanism enables IAVs to mutate continuously by point mutation. Reassortment refers to the exchange of genetic segments between IAVs inside coinfected cells. Both mechanisms contribute to the evasion of the immune response induced by previous influenza infections or by vaccination.

Wild waterfowl are believed to be the natural reservoir for IAVs^3^, although the transfer of IAVs from wild aquatic birds to domestic poultry is known to occur. After being introduced into poultry, low-pathogenic avian influenza (LPAI) H5 and H7 viruses have the potential to mutate into highly pathogenic avian influenza (HPAI) in domestic gallinaceous poultry through mechanisms that affect HA cleavage, such as through the insertion or substitution of basic amino acids at the HA0 cleavage site^4^ or by nonhomologous recombination of the HA gene with other viral genes or the host genome^5–7^. HPIAVs have caused devastating economic losses to the poultry industry and pose a serious threat to public health^8^.

H7 HPIAV strains have caused sporadic outbreaks in domestic poultry since they were first detected in 1995 and have become enzootic in domestic poultry in Pakistan^9,10^. From 1999 to 2004, epidemics caused by the H7N1 and H7N3 subtypes were reported in Italy^11,12^. In 2003, H7N7 HPIAV caused a severe outbreak in domestic poultry in the Netherlands that led to the culling of 30 million birds^13^. In the Americas, H7 HPIAVs have periodically emerged in poultry, with four outbreaks of H7N3 HPIAV occurring in Canada, Mexico, and Chile between 2002 and 2016, and there were also reports of H7N8 HPIAV in Indiana in 2016 and H7N9 HPIAV in Tennessee in 2017^6,7,14–17^.

Four genetically distinct strains of H7 LPIAVs were detected in domestic duck farms through active surveillance in the Republic of Korea in 2008–2011, which were observed to be closely related to viruses circulating in migratory birds^18^. In this study, we characterized IAV strains isolated from wild bird habitats in the Republic of Korea from 2010 to 2017. To expand our understanding of the genetic properties of H7 LPIAV and its pathogenesis, we performed phylogenetic and molecular-clock analyses and assessed the replication and pathogenic potential of representative H7 viruses in chickens.

## Results

### Isolation of avian H7 influenza viruses in the Republic of Korea

From January 2010 to March 2017, 700 IAVs were isolated from wild bird habitats in the Republic of Korea (Table 1). The proportion of H7 viruses isolated was 14.9%, and among the 104 H7 isolates, H7N7 viruses were the predominant subtype (72/104, 69.2%), followed by H7N9 (12/104, 11.5%), H7N1 (10/104, 9.6%), H7N2 (5/104, 4.8%), H7N3 (2/104, 1.9%), and H7N4, H7N6, and H7N8 (each 1/104, 1%). Until the end of 2016, the mean annual proportion of isolates belonging to the H7 subtype was 13.3%, with a range of 2.9–27.3%. However, in early 2017, 48 out of 110 IAVs (43.6%) isolated from wild aquatic birds were of the H7 subtype.

**Table 1:** Number of avian influenza viruses (AIVs) isolated from wild birds in the Republic of Korea between 2010 and March 2017

Year	No. of total samples	No. of AIV-positive samples (prevalence^a^; %)	No. of AIV-positive samples for H7 subtypes								
			H7N1	H7N2	H7N3	H7N4	H7N6	H7N7	H7N8	H7N9	Subtotal (proportion^b^; %)
2010	6789	56 (0.8)				1		2	1		4 (7.1)
2011	7156	48 (0.7)			1			1		11	13 (27.1)
2012	7889	42 (0.5)	5								5 (11.9)
2013	10,029	22 (0.2)	3					3			6 (27.3)
2014	13,228	137 (1.0)		3				1			4 (2.9)
2015	19,533	145 (0.7)						11			11 (7.6)
2016	9538	140 (1.5)						12		1	13 (9.3)
2017	3224	110 (3.4)	2	2	1		1	42			48 (43.6)
Sub-total	77,386	700 (0.9)	10	5	2	1	1	72	1	12	104 (14.9)

^a^[(Number of AIV-positive samples)/(number of total samples)] × 100 (%).

^b^[(Number of H7 viruses)/(number of AIV-positive samples)] × 100 (%).

### Molecular characteristics of H7 viruses

Representative isolates (*n* = 41) from among the 104 H7 isolates were selected for molecular characterization after considering geographical locations and collection dates (Supplementary table 1). We compared the molecular characteristics of whole-genome sequences using a Sanger sequencing platform. Most HA genes of the H7 isolates encoded PELPKGR/GLF sequences at the cleavage site (Supplementary table 2). However, A/mallard/Korea/H915/2017 (H7N7) viruses had different cleavage sites (PESPKGR/G). These motifs are associated with a low pathogenicity of IAV in chickens^19^. The amino acids at positions 177, 217, and 219 (H7 numbering) of the receptor-binding sites in the HA1 proteins indicated a preference for α2,3-linked sialic acid receptors rather than α2,6-linked sialic acid receptors^20^. Well-known mammalian adaptive markers^21^, such as E627K and D701N in PB2, were not detected in any of the H7 viruses, nor were specific amino acid substitutions at residue 66 in PB1-F2, residue 15 in M1, or residue 31 in M2. Most H7 isolates carried the P42S mutation in the NS1 protein, which has been shown to increase viral virulence in mice^22^.

### Genetic reassortment and viral evolution

**Fig. 1:**
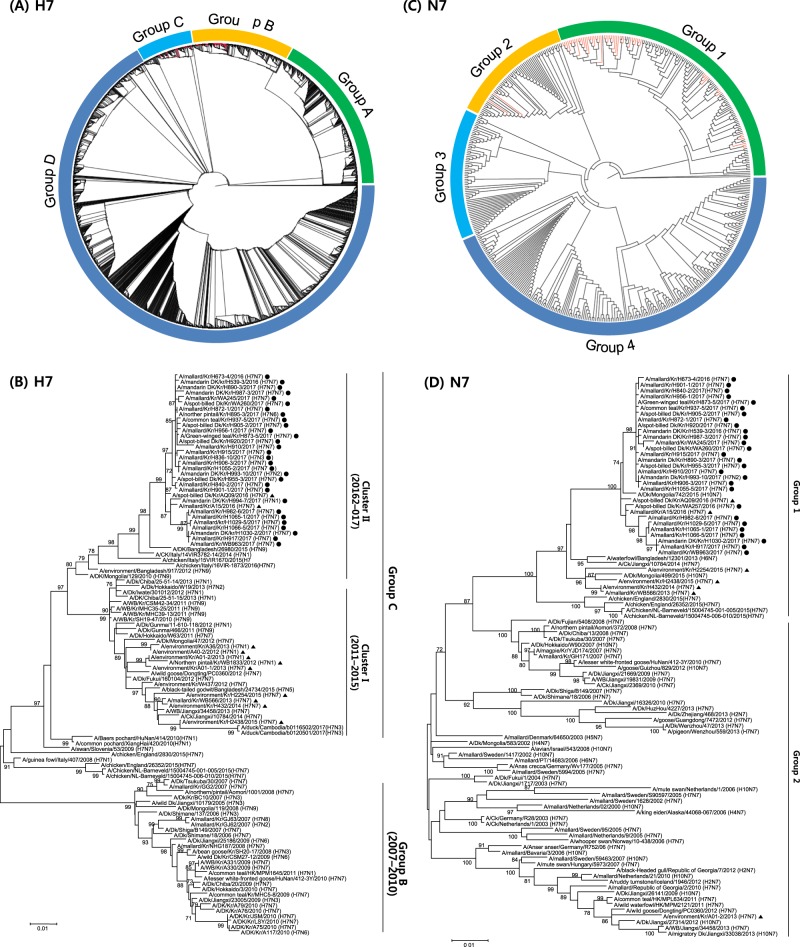
Phylogenetic analysis of H7 influenza A viruses in Eurasia. **a** Phylogenetic tree of the HA genes of 41 H7 influenza A viruses, representative of 107 viruses isolated in the Republic of Korea between 2010 and 2017 (red lines), in addition to all 2963 H7 sequence >1600 nt from viruses isolated in Eurasia that were available from the Global Initiative on Sharing All Influenza Data (GISAID). Evolutionary analyses were conducted in RAxML. Red lines denote the H7 viruses isolated in South Korea. **b** Maximum-likelihood phylogenetic tree of a subset of group B and C H7 genes. Tree stability was determined by bootstrap analysis with 1000 replicates, and bootstrap values >70% are displayed above the branch nodes. Evolutionary analyses were conducted in MEGA6. For the H7 viruses isolated in the Republic of Korea in this study, those isolated during 2016–2017 are indicated by dots, and those isolated at other times are indicated by triangles. Other viral sequences were obtained from GISAID. Ck chicken, Dk duck, Kr Korea, WBF wild bird feces. **c** Phylogenetic tree of NA genes of 33 H7N7 influenza A viruses isolated in the Republic of Korea between 2010 and 2017 (red lines) and all 505 N7 sequences >1400 nt from viruses isolated in Eurasia that were available from GISAID. Evolutionary analyses were conducted in RAxML. **d** Maximum likelihood phylogenetic tree of a subset of group 1 and 2 N7 genes. Tree stability was determined by bootstrap analysis with 1000 replicates, and bootstrap values >70% are displayed above the branch nodes. Evolutionary analyses were conducted in MEGA6. For the H7 viruses isolated in the Republic of Korea in this study, those isolated during 2016–2017 are indicated by dots, and those isolated at other times are indicated by triangles. Other viral sequences were obtained from GISAID. Ck chicken, Dk duck, Kr Korea, WBF wild bird feces

The HA genes of the H7 isolates were phylogenetically analyzed by the maximum-likelihood method together with available sequences from both the NCBI Influenza Virus Resource and Global Initiative on Sharing All Influenza Data (GISAID; http://www.gisaid.org). H7 genes of viruses isolated in Eurasia were phylogenetically divided into four groups (Fig. 1a). Nearly all of the H7 sequences from the isolates from the Republic of Korea fell within groups B and C, with the exception of one reference strain (A/wild bird feces/Hadoree/8/2003 (H7N3)) in group A. Otherwise, group A consisted of H7 viruses detected in Europe, and group D was composed of H7 viruses identified in China, including recently identified H7N9 viruses that were circulating in southern China and causing fatal infections in humans^23^. We performed further phylogenetic analyses focusing on H7 sequences of IAVs isolated in the Republic of Korea (Fig. 1b). Group B was composed of H7 viruses isolated in China, Japan, the Republic of Korea, and Thailand between 2007 and 2010. Group C could be divided into two genetic clusters, with cluster I comprising viruses isolated from wild birds and poultry in Asia from 2010 to 2015, while cluster II contained H7 viruses isolated between 2015 and 2017. Notably, cluster II of group C included the H7 gene from H7N7 HPIAV (A/chicken/Italy/16VIR-1873/2016 (H7N7)) that was detected in northeastern Italy in April 2016.

The NA phylogeny showed that N7 genes in the Eurasian lineage were also classified into four groups (Fig. 1c). All N7 sequences of the H7N7 viruses isolated in this study belonged to group 1, with the exception of one isolate (A/WBF/Kr/A01-2/2013 (H7N7)) in group 2 (Fig. 1d). Group 4 contained H7N7 viruses of poultry origin isolated in Europe, whereas other groups were composed of LPIAVs isolated from migratory birds in Eurasia.

**Fig. 2:**
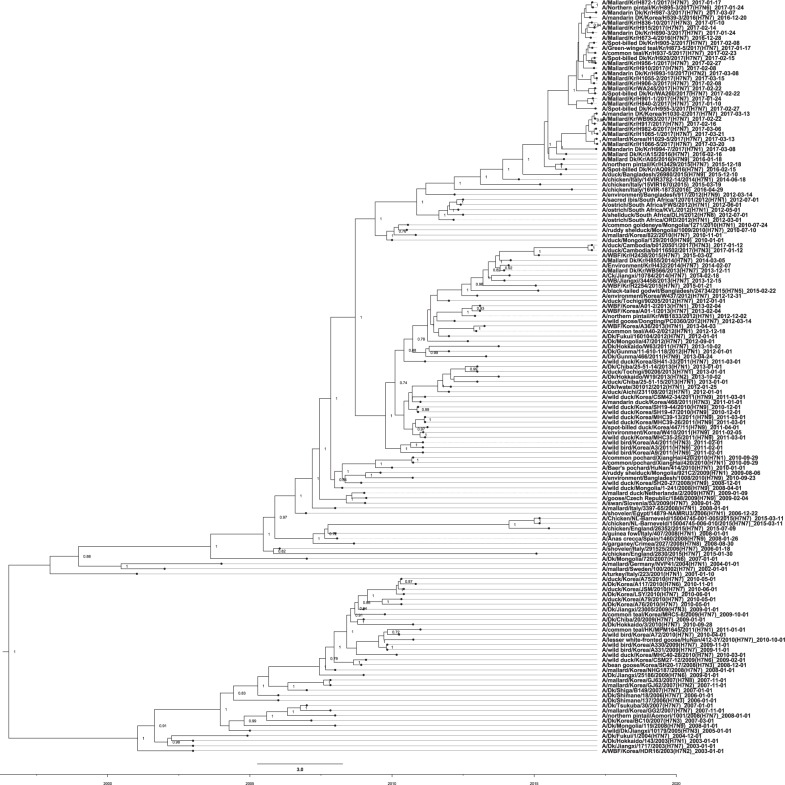
Evolution of avian influenza virus H7 subtypes in Eurasia. An HA nucleotide-sequence phylogeny was inferred using the SRD06 partitioned-substitution model, an uncorrelated lognormal relaxed clock, and a Bayesian skyline coalescent model in BEAST v1.8.1 with a chain length of 100 million. A genome-constellation analysis was performed by generating gene clusters for each segment using a 97% nucleotide identity cutoff. Cluster assignments are represented by one colored box for each gene segment, creating a genome constellation for each virus. Coloring between the columns is independent, and the total number of colors in a column reflects the number of clusters generated for that gene segment. Ck chicken, Dk duck, Kr Korea, WBF wild bird feces

To obtain a more detailed evolutionary history of the Korean H7 IAVs, we conducted molecular-clock analyses for the H7 gene segment using sequences from the Eurasian lineage. As shown in Fig. 2, the H7 viruses from the Republic of Korea were distinctly separated into groups B and C, consistent with the phylogenetic tree obtained by the maximum-likelihood algorithm. The time-scaled maximum clade credibility (MCC) phylogeny indicated that group B was no longer present in the Republic of Korea after 2010, having been replaced with group C, which evolved into two genetically distinct clusters. The estimated time to the most recent common ancestor was October 2009 (95% highest posterior density interval (HPD), April 2009 to March 2010) for cluster I and May 2009 (95% HPD, November 2008–October 2009) for cluster II. A putative common ancestor of cluster II H7 viruses isolated in the Republic of Korea was estimated to have diverged between January 2014 and February 2015 (95% HPD). These novel H7 viruses in cluster II were further divided into two subgroups in approximately June 2015 (95% HPD, February–October 2015).

**Fig. 3:**
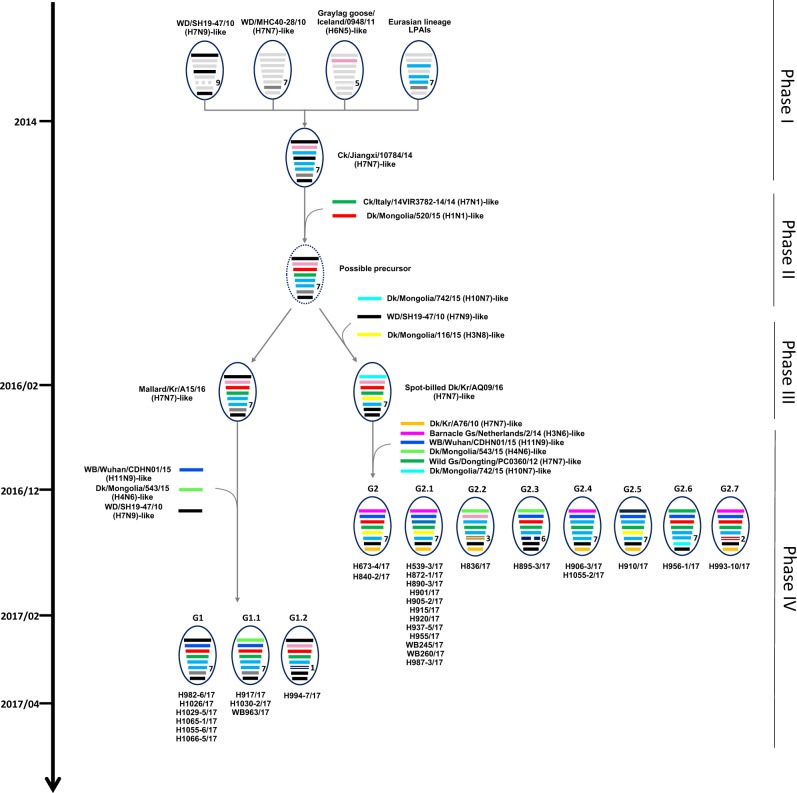
Proposed evolutionary pathways leading to the generation of novel-reassortant H7 viruses. Influenza A viruses are represented by ovals containing horizontal bars for the eight gene segments (from top to bottom, PB2, PB1, PA, HA, NP, NA, MP, and NS). Different colors represent different viral lineages. Solid ovals represent virus strains isolated from wild and domestic birds, and dotted ovals represent hypothetical viral strains. The timescale is indicated on the left side, and the different phases of evolution are indicated on the right side. The labels below the viral ovals are identifiers for corresponding viruses isolated in this study. Ck chicken, Dk duck, Kr Korea, WBF wild bird feces, Gs goose

**Fig. 4:**
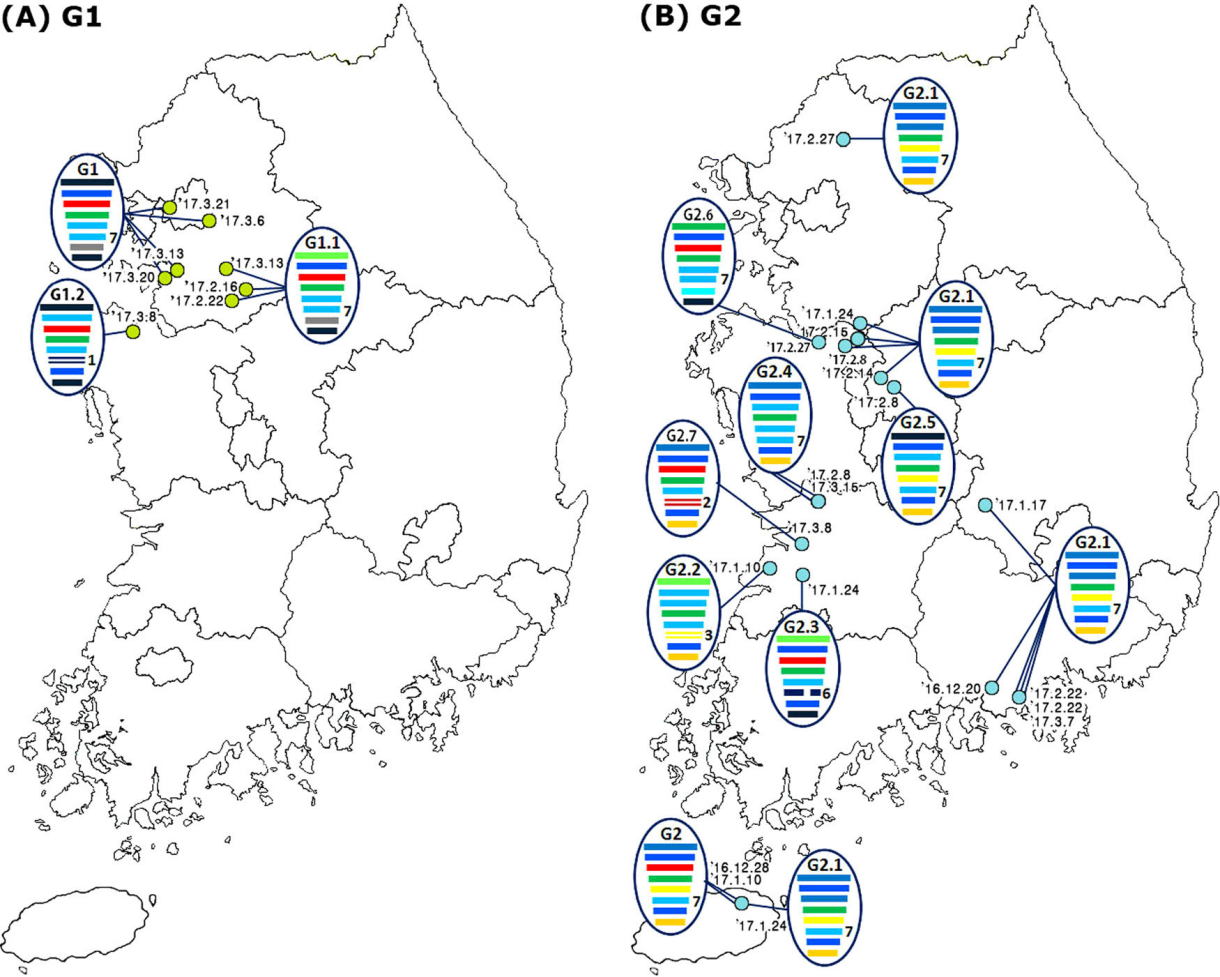
Locations of isolation of H7-subtype low pathogenicity (LP) influenza A viruses (IAVs) from wild bird habitats in the Republic of Korea during the winter of 2016–2017. The light green and blue circles indicate the locations where the G1 (**a**) and G2 (**b**) subgroups of the H7 LPIAVs were isolated from wild bird habitats, and the associated collection dates are indicated. The IAVs are represented by ovals containing horizontal bars for the eight gene segments (from top to bottom, PB2, PB1, PA, HA, NP, NA, MP, and NS). The different colors represent different virus lineages. Genotypes G1 and G2 were subgroups within cluster II of group C of H7 LPIAVs

A genomic constellation analysis in which each segment was clustered at a 97% nucleotide identity cutoff demonstrated that the H7 viruses isolated in the Republic of Korea could be differentiated into ≥20 different genotypes until early 2015 (Fig. 2). From late 2015 onwards, ≥13 additional different genotypes of novel-reassortant H7 viruses in cluster II were identified in migratory birds in the Republic of Korea (Fig. 3). These reassortant viruses were classified into subgroups G1 and G2 according to the divergence in the molecular-clock phylogeny of the HA gene (Fig. 2). The G1 subgroup comprised viruses isolated from the feces of mallard or mandarin ducks in Seoul, Gyeonggi, and Chungnam provinces from February to March 2017 (Fig. 4a). The G1.1 and G1.2 genotypes differed from that of the G1 genotype by the inclusion of the PB2 (G1.1) or PB1, NA, and MP (G1.2) segments from Eurasian LPIAVs. The G2 subgroup was composed of viruses recovered from the feces of various wild bird species on a national scale from December 2016 to March 2017 (Fig. 4b). The genotypes of seven G2-derived isolates (G2.1–G2.7) were reassortants with Eurasian LPIAVs.

### Replication and transmission of novel H7N7 viruses in chickens

To determine the replication and transmission capacities of two novel H7N7 viruses (A/mandarin duck/Korea/H539-3/2016 (H7N7), H539-3, the representative strain of the G2.1 genotype, and A/mallard/Kr/H982-6/2017 (H7N7), H982-6, the representative strain of the G1 genotype) in chickens were used to assess oropharyngeal and cloacal viral shedding in both virus-challenged and direct-contact groups, which was monitored for 14 days p.i. (Table 2, Supplementary table 3). None of the challenged or directly contacted chickens exhibited clinical signs. Nevertheless, one of five chickens challenged with the H539-3 virus died without clinical signs at day 8 p.i. In chickens challenged with H539-3, viral shedding in oropharyngeal and cloacal swabs in the challenged and direct-contact groups began as early as day 3 p.i. In the H539-3-challenged group, the maximal viral loads in oropharyngeal swabs (2.0 ± 1.6 log_10_EID_50_/ml) were reached at day 3 p.i., and these viral loads gradually decreased to day 7 p.i. By contrast, the peak of cloacal shedding (4.2 ± 2.5 log_10_EID_50_/ml) occurred at day 7 p.i., while the viral shedding lasted until day 14 p.i. In the H539-3 direct-contact group, two peaks of viral shedding were observed in oropharyngeal swabs: in two of three chickens, the peak occurred on day 5 p.i. (1.9 ± 2.0 log_10_EID_50_/ml), while the peak occurred on day 10 p.i. (1.4 ± 2.45 log_10_EID_50_/ml) for the other chicken. Viral shedding in cloacal swabs peaked on day 7 p.i. (3.5 ± 3.2 log_10_EID_50_/ml). All challenged and direct-contact chickens in the H539-3 group had undergone seroconversion by day 14 p.i., as determined by hemagglutination inhibition with a homologous HA antigen. By contrast, we did not detect any virus in the oropharyngeal or cloacal swabs from challenged or direct-contact chickens in the H982-6 group, and there no seroconversion was observed in these chickens. These results indicated that the H982-6 virus was not yet well adapted to chickens.

**Table 2:** Replication and transmission of representative H7N7 viruses in 4-week-old specific-pathogen-free chickens

Isolate	Group	Sample	Swab viral titers (log_10_EID_50_/ml)^a^					Seroconversion (HI titer)^b^
			3 dpi	5 dpi	7 dpi	10 dpi	14 dpi	
A/mandarin Dk/Kr/H539-3/2016 (H7N7)	Challenged	OP	4/5 (2.0 ± 1.6)	3/5 (1.8 ± 1.7)	1/5 (0.2 ± 0.5)	0/4	0/4	4/4 (64, 256, 256, 8)
		CL	3/5 (2.2 ± 2.4)	4/5 (3.6 ± 2.6)	4/5 (4.2 ± 2.5)	2/4 (1.6 ± 2.0)	2/4 (1.6 ± 2.5)	
	Direct contact	OP	2/3 (1.0 ± 1.1)	2/3 (1.9 ± 2.0)	0/3	1/3 (1.4 ± 2.4)	1/3 (0.4 ± 0.7)	3/3 (8, 16, 32)
		CL	1/3 (0.7 ± 1.2)	1/3 (1.4 ± 2.4)	2/3 (3.5 ± 3.2)	1/3 (1.4 ± 2.4)	1/3 (1.7 ± 3.0)	
A/mallard/Kr/H982-6/2017 (H7N7)	Challenged	OP	0/5	0/5	0/5	0/5	0/5	0/5
		CL	0/5	0/5	0/5	0/5	0/5	
	Direct contact	OP	0/3	0/3	0/3	0/3	0/3	0/3
		CL	0/3	0/3	0/3	0/3	0/3	

*CL* cloacal, *dpi* days post-infection, *EID*_*50*_ 50% egg infective dose, *Dk* duck, *HI* hemagglutination inhibition, *Kr* Korea, *OP* oropharyngeal

Values shown are (number of infected birds)/(number of inoculated birds) in the challenge group, and (number of infected birds)/(number of naïve birds) in the direct-contact group. Values in parentheses are virus titer (log_10_EID_50_/ml), mean ± standard deviation. The inoculation dose for chickens was 10^6^ EID_50_/0.1 ml

^a^Mean viral titers from OP and CL swabs were calculated by the Reed and Muench method.

^b^Sera were collected from chickens at 14 dpi. Seroconversion was confirmed by HI assay.

To determine whether the H7 representative viruses were efficiently replicated in chickens, we attempted to isolate viruses from several types of tissue obtained from the challenged chickens at day 4 p.i. The H539-3 virus replicated in the tracheas (2.2 ± 1.5 log_10_EID_50_/ml), cecal tonsils (3.1 ± 2.1 log_10_EID_50_/ml), and pancreases (4.4 ± 3.2 log_10_EID_50_/ml) of three out of four chickens (Table 3). Moreover, the H539-3 virus was also detected in the lungs (1.6 ± 1.9 log_10_EID_50_/ml), kidneys (2.9 ± 3.3 log_10_EID_50_/ml), spleens (1.4 ± 1.6 log_10_EID_50_/ml), and brains (1.6 ± 1.8 log_10_EID_50_/ml) of two out of four chickens. However, no virus was detected in the tissues of the H982-6-challenged chickens.

**Table 3:** Virus titers in tissues from 4-week-old specific-pathogen-free chickens challenged with representative H7N7 isolates

Isolate	Tissue						
	Trachea	CT	Lung	Kidney	Spleen	Brain	Pancreas
A/mandarin Dk/Kr/H539-3/2016 (H7N7)	3/4 (2.2 ± 1.5)	3/4 (3.1 ± 2.1)	2/4 (1.6 ± 1.9)	2/4 (2.9 ± 3.3)	2/4 (1.4 ± 1.6)	2/4 (1.6 ± 1.8)	3/4 (4.4 ± 3.2)
A/mallard/Kr/H982-6/2017 (H7N7)	0/3	0/3	0/3	0/3	0/3	0/3	0/3

*EID*_*50*_ 50% egg infective dose, *CT* cecal tonsil, *Dk* duck, *Kr* Korea

Values shown are (number of birds with virus in the tissue)/(number of inoculated birds). Values in parentheses are virus titer (log_10_EID_50_/g). The virus titer is the mean ± standard deviation of the samples. The inoculation dose for chickens was 10^6^ EID_50_/0.1 ml

### Histopathological examination

**Fig. 5:**
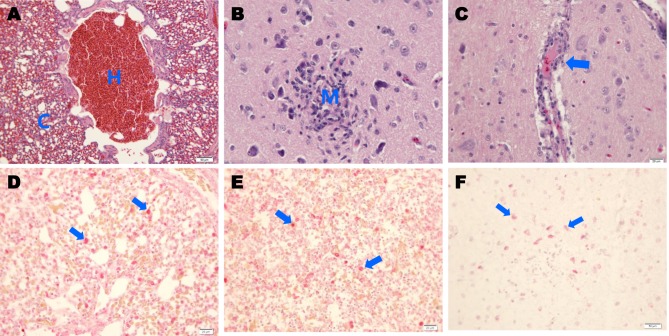
Histopathological changes and immunostaining of tissues infected with A/mandarin duck/Korea/H539-3/2016 (H7N7). Photomicrographs of hematoxylin and eosin (H&E)-stained and immunohistochemically stained tissue sections collected 4 days postinfection from chickens challenged with A/mandarin duck/Korea/H539-3/2016 (H7N7) virus. **a** Chickens challenged with the H7N7 virus showing severe hemorrhaging (**h**) and congestion (**c**) in lung tissue (H&E stain). **b** Infected chickens showing focal necrosis and mononuclear-cell infiltration (**m**) in cerebral tissue (H&E stain). **c** Infected chickens showing mild perivascular cuffing (arrow) in cerebral tissue (H&E stain). **d** IAV NP antigens were immunostained in alveolar macrophages (arrows) in lung tissue. **e** IAV antigens were immunostained in macrophages of red pulp (arrows) in spleen tissue. **f** IAV antigens were immunostained in cerebral tissue neurons (arrows)

Histopathological lesions were prominently observed in the lung and brain tissues of the H539-3-challenged chickens (Fig. 5). Moderate diffuse congestion and hemorrhaging were observed in the lungs (Fig. 5a), and focal malacia with deposition of mononuclear cells (Fig. 5b) and mild perivascular cuffing (Fig. 5c) were observed in the cerebra. IAV antigens were present in alveolar macrophages (Fig. 5d) and in macrophages from the spleens of H539-3-challenged animals (Fig. 5e). In addition, viral antigens were detected in the neurons of necrotic areas in the cerebra (Fig. 5f).

## Discussion

In this study, we genetically characterized 41 H7 LPIAVs isolated from wild bird habitats in the Republic of Korea from 2012 to 2017. Notably, H7 subtypes accounted for almost half of IAVs detected (48 of 110) from wild birds in early 2017 (Table 1). The timeline and geographical distribution of the isolates (Fig. 4) and the results from molecular-clock analysis (Fig. 2) suggested that the viruses belonging to the G1 and G2 subgroups were separately introduced via different migratory bird populations. Migratory birds moving within several flyways in Eurasia exhibit overlap in the locations of circumpolar arctic and subarctic breeding areas, with some sharing common breeding areas^24^. The identification of H7 IAV infections in migratory birds in the Republic of Korea via a rigorously implemented and systematic national IAV active-surveillance program indicates the potential for dissemination of these novel-reassortant group C H7 viruses throughout Eurasia.

The consequences of LPAI viruses of wild bird origin (such as H7N1^11^ and H7N7^13^) mutating into HPIAVs after their introduction and replication in poultry have been repeatedly demonstrated. In April 2016, a H7N7 HPIAV was detected on an organic free-range hen farm in northeastern Italy^25^. Epidemiological data, supported by the presence of serologically positive birds and the results of phylogenetic analysis, revealed that the virus was probably introduced from migratory birds as a LPAI through direct contact^25^. In this study, we showed that the HA gene of this virus (A/chicken/Italy/16vir1873/2016 (H7N7)) belongs to cluster II of group C of H7 IAVs, which includes the H7 IAVs isolated in 2016 and early 2017 in the Republic of Korea. Unfortunately, sequence information for other segments of this virus has not been published. However, considering the HPAI outbreak that was caused by A/chicken/Italy/16vir1873/2016 (H7N7) in Italy in 2016, the unusually high prevalence of H7 viruses belonging to cluster II of group C in wild bird habitats during the winter of 2016–2017 raises concerns of further outbreaks in poultry by this viral lineage.

European avian H7N7 strains have been reported to not induce clinical signs in chickens, despite substantial replication in the respiratory tracts and brains of challenged chickens and to be rapidly transmitted to chickens through direct contact^26^. Notably, we observed that the H539-3 virus (the representative strain of the G2.1 genotype) replicated systemically in chickens, as indicated by the recovery of the virus from various tissues, including the brain, without clinical signs except mortality (in one animal). PCR cloning of the targeted cleavage site and Sanger sequencing analyses showed that viruses isolated from the organs of H539-3-challenged chickens retained a monobasic HA cleavage site, characteristic of LPIAV (data not shown). However, in a previous study, a small proportion of the viruses from samples of turkeys challenged with A/chicken/Italy/1279/99 (H7N1) LPIAV were observed to exhibit a molecular signature of HPIAV at the HA cleavage site using ultradeep amplicon sequencing with a Next Generation Sequencing (NGS) platform^27^. Therefore, the swab samples and tissues that we collected from chickens challenged with the H539-3 virus will be further analyzed for the presence of a molecular signature of HPIAV using ultradeep amplicon sequencing with an NGS system to investigate the potential of evolution from LPIAV to HPIAV over the course of the infection. Similarly, further research on the serial passage of LPIAV by direct contact in chickens is required to determine whether the virus could acquire multiple basic amino acids at the HA cleavage site under natural conditions.

On the basis of the available evidence, we propose an evolutionary mechanism that could have led to the generation of novel H7 LPIAVs. The H7N7 precursor virus could have been generated in migratory birds through four phases of sequential reassortment events (Fig. 3). The first phase of reassortment may have occurred among the group B H7 viruses and other LPIAVs circulating in Eurasian migratory birds from 2010 to 2014, generating an A/chicken/Jiangxi/10784/2014 (H7N7)-like virus. In the second phase, further reassortment between the A/chicken/Jiangxi/10784/2014-like virus, which possesses six H7N7-like gene segments (PB2, PB1, NP, NA, MP, and NS), and Eurasian LPIAVs in wild bird populations with the HA gene of cluster II of group C and the PA gene present in A/duck/Mongolia/520/2015 (H1N1) could have led to the generation of a ‘possible-precursor’ virus. In February 2016, two LPIAV genotypes emerged in the Republic of Korea. A/mallard/Korea/A15/2016 (H7N7), or 16A15, was estimated to be most closely related to the possible-precursor virus, whereas A/spot-billed duck/Korea/AQ09/2016 (H7N7), or 16AQ09, contained a possible-precursor backbone with novel PB2, NP, and MP gene segments. The fourth phase of reassortment may have independently involved both 16A15-like and 16AQ09-like viruses. The H982-6-like viruses, which possess seven genes derived from 16A15-like viruses as well as the PB1 gene from an A/wild bird/Wuhan/CDHN01/2015 (H11N9)-like virus, were detected in early 2017 and designated as G1-genotype viruses. In late 2016, A/mallard/Korea/H673-4/2017 (H7N7)-like viruses, which possess the 16AQ09-like backbone with four genes derived from Eurasian LPIAVs, were isolated and designated as G2-genotype viruses. Moreover, both the G1 and G2 genotypes had diversified via genetic reassortment with other LPIAVs circulating in wild bird populations in Eurasia, producing at least nine related genotypes (G1.1–G1.2 and G2.1–G2.7). Regarding the generation time of the possible-precursor virus, we observed that cluster II H7 viruses divided into two subgroups in approximately June 2015, coinciding with the breeding season. Therefore, it was likely that migratory birds had carried possible-precursor viruses during their southern migration in 2015 and when they returned to the breeding grounds. The high prevalence of novel H7 reassortants during the winter of 2016–2017 likely reflects the introduction of viruses into a naïve population of migratory birds with no previous exposure to viruses from cluster II of group C. Thus, novel H7 reassortant viruses seem to have undergone further genetic reassortment with other LPIAVs and spread between migratory bird species in these breeding sites prior to their subsequent dissemination during the 2016 southern-migration period.

The existence of triple-reassortant internal gene constellations (involving gene segments from human, swine, and avian IAV types) has been well established in multiple viral subtypes among North American swine, suggesting that they have a selective advantage over other constellations^28^. In contrast to a limited number of stable genome constellations in mammalian-adapted IAV types, IAVs have been suggested to continuously form diverse and transient gene constellations in migratory birds through reassortment^29^. Nevertheless, the persistence of specific gene constellations in wild bird populations was previously associated with the emergence of a novel H7N8 HPIAV in North America^30^. We observed that the H539-3 and H982-6 viruses, representative strains of novel H7 reassortants, displayed remarkable differences in pathogenesis in chickens, even though we believe that they evolved from the same precursor virus. from the results of several studies evaluating reassortant viruses demonstrate that virus pathogenicity is dependent on the functional integrity of each gene and on the generation of a genome configuration that is optimal for the infection of a given host^31,32^. Therefore, the identification of genetic markers or gene constellations responsible for the pathogenesis, adaptation, and transmission of novel H7 reassortants in chickens should be an important objective for future studies.

In summary, this investigation has provided information on the geographical distribution, representation, genetic diversity, possible evolutionary pathways, and pathogenic potential of novel H7 reassortants in chickens isolated from wild bird habitats in the Republic of Korea. H7 viruses in cluster II of group C with considerable genetic diversity were detected at an unusually high frequency in the winter of 2016–2017, and some of these viruses were capable of replicating systemically and transmitting efficiently in chickens without prior adaptation, as evidenced by virus isolation and histopathological examination. The efficient subclinical replication and shedding of viruses in chickens may facilitate their silent spread among poultry. Thus, the recent appearance of novel H7 reassortants in migratory bird habitats highlights the need for continued influenza surveillance in both wild birds and poultry in Eurasia.

## Materials and methods

### Sample collection and virus isolation

During the national surveillance programs in the Republic of Korea between January 2010 and March 2017, 77,386 samples were collected from wild bird habitats. Sample collection was performed by the Livestock Health Control Association or by regional veterinary offices. Fresh fecal samples and bird carcasses were collected in major migratory habitats. In addition, some migratory birds in these habitats were captured by cannon netting, and oropharyngeal and cloacal swabs were collected from these animals.

Oropharyngeal, cloacal, and fecal samples, as well as tissue homogenates from dead birds, were suspended in phosphate-buffered saline containing gentamicin and inoculated into the allantoic cavities of 9–11-day-old specific-pathogen-free (SPF) embryonated eggs. After a 96-h incubation at 37 °C, the eggs were chilled, and allantoic fluids were subsequently harvested and tested for hemagglutination activity using chicken erythrocytes. A barcoding system utilizing mitochondrial DNA from wild bird feces was used to determine the host species of IAVs isolated from wild birds, as previously described^33^.

### Viral sequencing and phylogenetic analysis

Viral RNA was extracted from allantoic fluid using a Patho Gene-Spin Viral DNA/RNA extraction kit (Intron Biotechnology, Seongnam, South Korea). The viral HA and NA subtypes were characterized by reverse transcription (RT)-PCR. All viral RNA segments of the H7 isolates were amplified using universal and gene-specific primers with an Omniscript reverse transcription kit (QIAGEN, Germantown, MD, USA) and an Ex Taq polymerase (TAKARA, Kusatsu, Japan) and sequenced with an ABI 3730xl DNA analyzer (Applied Biosystems, CA, USA)^34^.

Representative IAV sequences from other countries used in the phylogenetic comparison were obtained from the NCBI Influenza Virus Resource (https://www.ncbi.nlm.nih.gov/genomes/FLU/Database/nph-select.cgi?go=database) and the GISAID. For the H7 and N7 genes, all of the data available from GISAID were used. The sequences of these genes were aligned using the MAFFT tool^35^, and phylogenetic trees for these gene segments were constructed by the maximum-likelihood method in the online version of RAxML (v8.2.10). Gene-segment-specific phylogenetic trees were also generated using the maximum-likelihood method implemented in MEGA (version 6.0). A general time-reversible model of nucleotide substitution with gamma distribution and invariant sites was applied throughout the analyses.

### Molecular clock analysis

The time-scaled phylogenies, nucleotide-substitution rates, and mean estimated times to most-recent common ancestor were estimated by the Bayesian Markov chain Monte Carlo method implemented in BEAST v1.8.1^36^. Genomic sequences with complete sampling dates (exact month, day, and year) were selected for the HA genes as the basis of the phylogenetic trees. The SRD06 partitioned-substitution model and uncorrelated lognormal relaxed-clock model were used, with a Bayesian skyline coalescent tree prior. Two independent runs were performed, with a chain length of 100 million steps sampled every 10,000 steps. Outputs and effective sample size were examined with Tracer v1.6 (http://tree.bio.ed.ac.uk/software/tracer/). The outputs were combined with a 10% burn-in, and an MCC tree was summarized with TreeAnnotator v1.8.0 (http://beast.bio.ed.ac.uk/TreeAnnotator/) and edited in FigTree v1.4.0 (http://tree.bio.ed.ac.uk/software/figtree/).

### Genotyping

The genotypes were defined on the basis of the gene-segment-specific phylogenetic trees for the H7 isolates. A monophyletic group was identified by two criteria: it was supported by a bootstrap value >70, and all sequences in the group had nucleotide-sequence identities >97%. Each genotype was the combination of the cluster assignment of eight gene segments.

### Animal experiments

Representative viruses of two dominant H7 isolate genotypes were intranasally inoculated into 4-week-old SPF white leghorn chickens using 0.1 ml of a 10^6^ 50% egg infectious dose (EID_50_). The accuracy of the viral challenge doses inoculated was confirmed by immediate back titration in SPF embryonated eggs. To investigate potential virus transmission by direct contact, three infection-naïve chickens were cohoused with the inoculated chickens 8 h after inoculation. Oropharyngeal and cloacal swabs were collected on days 3, 5, 7, 10, and 14 postinfection (p.i.). To determine the patterns of viral replication in the major tissues of the inoculated birds, chickens were euthanized on day 4 p.i. For each animal, trachea, cecal tonsil, lung, spleen, kidney, brain, and pancreas tissue samples were collected, homogenized, and centrifuged at 1400×*g* at 4 °C for 10 min. The supernatants were serially diluted 10-fold and inoculated into 10–11-day-old embryonated chicken eggs. After 3 days of incubation at 37 °C, the eggs were chilled, and the allantoic fluid harvested from each egg was tested for hemagglutinin activity. The virus titer of each sample was calculated by the Reed–Muench method^37^. In addition, monitoring of viral shedding in challenged animals was also carried out by matrix gene-specific TaqMan real-time RT-PCR (rRT-PCR) assay^38^. Sera collected from each remaining chicken on day 14 p.i. were tested via a hemagglutination inhibition assay according to the International Epizootic Office (OIE) recommendations. All experiments were performed in a biosafety level 2 facility at the Animal and Plant Quarantine Agency (APQA), according to the guidelines of the Institutional Animal Care and Use Committee of the APQA in the Republic of Korea.

### Histopathology and immunohistochemistry

On day 4 p.i., parenchymal tissues were collected for histopathological analysis. Collected tissues were fixed for 24 h in 10% buffered neutral formaldehyde and then were processed for paraffin embedding. Paraffin sections were cut into 5-µm-thick sections, dewaxed, and then stained with hematoxylin and eosin. Duplicate sections were used in immunohistochemical analyses to assess the distribution of influenza viral antigens in individual tissues. Briefly, sections were stained with a mouse monoclonal antibody against influenza A virus nucleoprotein (MCA-400, AbD Serotec, Duesseldorf, Germany) and then were treated with a biotinylated goat anti-mouse-IgG secondary antibody. Bound antibodies were detected with an avidin-biotin detection system (Ventana Medical Systems, Tucson, AZ, USA). The chromogenic substrate was provided by the RedMap kit (Ventana Medical Systems).

## Electronic supplementary material

Supplementary table 1 and 2 and 3
