# Supplementary material for: Pathogenesis and genetic characteristics of novel reassortant low-pathogenic avian influenza H7 viruses isolated from migratory birds in the Republic of Korea in the winter of 2016–2017
Source: Emerg Microbes Infect. 2018 Nov 15;7:182. doi: 10.1038/s41426-018-0181-3 (PMC6237977; doi:10.1038/s41426-018-0181-3)
Supplement: Supplementary file 1 — Supplementary table 1 and 2 and 3 [file 41426_2018_181_MOESM1_ESM.doc]

0Supplementary table 1. H7 isolates used in this study (*n* = 41)

| Genotype | Virus name | Subtype | Collection date | Region | Samples | GISAID Isolate ID |
| --- | --- | --- | --- | --- | --- | --- |
| G1 | A/mallard/Korea/H982-6/2017 | H7N7 | 2017-03-06 | Seoul | Feces | EPI_ISL_309201 |
|  | A/mallard/Korea/H1029-5/2017 | H7N7 | 2017-03-13 | GG | Feces | EPI_ISL_309202 |
|  | A/mallard/Korea/H1065-1/2017 | H7N7 | 2017-03-21 | Seoul | Feces | EPI_ISL_309203 |
|  | A/mallard/Korea/H1066-5/2017 | H7N7 | 2017-03-20 | GG | Feces | EPI_ISL_309204 |
| G1.1 | A/mallard/Korea/H917/2017 | H7N7 | 2017-02-16 | GG | Feces | EPI_ISL_309205 |
|  | A/mandarin duck/Korea/H1030-2/2017 | H7N7 | 2017-03-13 | GG | Feces | EPI_ISL_309206 |
|  | A/mallard/Korea/WB963/2017 | H7N7 | 2017-02-22 | GG | OP, CL | EPI_ISL_309207 |
| G1.2 | A/mandarin duck/Korea/H994-7/2017 | H7N1 | 2017-03-08 | CN | Feces | EPI_ISL_309208 |
| G2 | A/mallard/Korea/H673-4/2016 | H7N7 | 2016-12-28 | JJ | Feces | EPI_ISL_309209 |
|  | A/mallard/Korea/H840-2/2017 | H7N7 | 2017-01-10 | JJ | Feces | EPI_ISL_309210 |
| G2.1 | A/mandarin duck/Korea/H539-3/2016 | H7N7 | 2016-12-20 | GN | Feces | EPI_ISL_309211 |
|  | A/mallard/Korea/H872-1/2017 | H7N7 | 2017-01-17 | GB | Feces | EPI_ISL_309212 |
|  | A/green-winged teal/Korea/H873-5/2017 | H7N7 | 2017-01-17 | CN | Feces | EPI_ISL_309213 |
|  | A/mandarin duck/Korea/H890-3/2017 | H7N7 | 2017-01-24 | GG | Feces | EPI_ISL_309214 |
|  | A/mallard/Korea/H901-1/2017 | H7N7 | 2017-01-24 | JJ | Feces | EPI_ISL_309215 |
|  | A/spot-billed duck/Korea/H905-2/2017 | H7N7 | 2017-02-08 | CN | Feces | EPI_ISL_309216 |
|  | A/mallard/Korea/H915/2017 | H7N7 | 2017-02-14 | CB | Feces | EPI_ISL_309217 |
|  | A/spot-billed duck/Korea/H920/2017 | H7N7 | 2017-02-15 | CN | Feces | EPI_ISL_309218 |
|  | A/common teal/Kr/H937-5/2017 | H7N7 | 2017-02-23 | CN | Feces | EPI_ISL_309219 |
|  | A/spot-billed duck/Korea/H955-3/2017 | H7N7 | 2017-02-27 | GG | Feces | EPI_ISL_309241 |
|  | A/mallard/Korea/WA245/2017 | H7N7 | 2017-02-22 | GN | OP, CL | EPI_ISL_309220 |
|  | A/spot-billed duck/Korea/WA260/2017 | H7N7 | 2017-02-22 | GN | OP, CL | EPI_ISL_309221 |
|  | A/mandarin duck/Korea/H987-3/2017 | H7N7 | 2017-03-07 | GN | Feces | EPI_ISL_309222 |
| G2.2 | A/mallard/Korea/H836-10/2017 | H7N3 | 2017-01-10 | JB | Feces | EPI_ISL_309223 |
| G2.3 | A/norther pintail/Korea/H895-3/2017 | H7N6 | 2017-01-24 | JB | Feces | EPI_ISL_309224 |
| G2.4 | A/mallard/Korea/H906-3/2017 | H7N7 | 2017-02-08 | JB | Feces | EPI_ISL_309225 |
|  | A/mallard/Korea/H1055-2/2017 | H7N7 | 2017-03-15 | JB | Feces | EPI_ISL_309226 |
| G2.5 | A/mallard/Korea/H910/2017 | H7N7 | 2017-02-08 | CB | Feces | EPI_ISL_309227 |
| G2.6 | A/mallard/Korea/H956-1/2017 | H7N7 | 2017-02-27 | CN | Feces | EPI_ISL_309228 |
| G2.7 | A/mandarin duck/Korea/H993-10/2017 | H7N2 | 2017-03-08 | JB | Feces | EPI_ISL_309229 |
| unclassified | A/northern pintail/Korea/WB1833/2012 | H7N1 | 2012-12-02 | JB | Feces | EPI_ISL_309230 |
|  | A/environment/Korea/A40-2/2012 | H7N1 | 2012-12-18 | JJ | Feces | EPI_ISL_309231 |
|  | A/environment/Korea/A01-1/2013 | H7N1 | 2013-02-04 | JJ | Feces | EPI_ISL_309232 |
|  | A/environment/Korea/A01-2/2013 | H7N7 | 2013-02-04 | JJ | Feces | EPI_ISL_309233 |
|  | A/environment/Korea/A36/2013 | H7N1 | 2013-04-02 | GW | Feces | EPI_ISL_309234 |
|  | A/mallard/Korea/WB566/2013 | H7N7 | 2013-12-11 | Busan | Feces | EPI_ISL_309235 |
|  | A/environment/Korea/H432/2014 | H7N7 | 2014-02-07 | JB | Feces | EPI_ISL_309236 |
|  | A/environment/Korea/H2254/2015 | H7N7 | 2015-01-21 | GG | Feces | EPI_ISL_309237 |
|  | A/environment/Korea/H2438/2015 | H7N7 | 2015-03-02 | GG | Feces | EPI_ISL_309238 |
|  | A/mallard/Korea/A15/2016 | H7N7 | 2016-02-16 | GG | Feces | EPI_ISL_309239 |
|  | A/spot-billed duck/Korea/AQ09/2016 | H7N7 | 2016-02-15 | Ulsan | Feces | EPI_ISL_309240 |

GISAID, Global Initiative on Sharing All Influenza Data; GG, Gyeonggi-do; CN, Chungcheongnam-do; JJ, Jeju-do; GN, Gyeongsangnam-do; GB, Gyeongsangbuk-do; CB, Chungcheongbuk-do; OP, oropharyngeal; CL, cloacal; JB, Jeollabuk-do; GW, Gangwon-do.

Supplementary table 2. Molecular characteristics associated with viral pathogenicity of H7 isolates

| Virus | Subtype | HA^a^ | | | |  | PB2 | |  | PB1-F2 |  | M1 |  | M2 |  | NS1 |
| --- | --- | --- | --- | --- | --- | --- | --- | --- | --- | --- | --- | --- | --- | --- | --- | --- |
|  |  | Cleavage site | 177 | 217 | 219 |  | 627 | 701 |  | 66 |  | 15 |  | 31 |  | 42 |
| A/mallard/Kr/H982-6/2017 | H7N7 | PELPKGR↓GLF | G | Q | G |  | E | D |  | N |  | V |  | S |  | S |
| A/mallard/Kr/H1029-5/2017 | H7N7 | PELPKGR↓GLF | G | Q | G |  | E | D |  | N |  | V |  | S |  | S |
| A/mallard/Kr/H1065-1/2017 | H7N7 | PELPKGR↓GLF | G | Q | G |  | E | D |  | N |  | V |  | S |  | S |
| A/mallard/Kr/H1066-5/2017 | H7N7 | PELPKGR↓GLF | G | Q | G |  | E | D |  | N |  | V |  | S |  | S |
| A/mallard/Kr/H917/2017 | H7N7 | PELPKGR↓GLF | G | Q | G |  | E | D |  | N |  | V |  | S |  | S |
| A/mandarin DK/Kr/H1030-2/2017 | H7N7 | PELPKGR↓GLF | G | Q | G |  | E | D |  | N |  | V |  | S |  | S |
| A/mallard/Kr/WB963/2017 | H7N7 | PELPKGR↓GLF | G | Q | G |  | E | D |  | N |  | V |  | S |  | S |
| A/mandarin DK/Kr/H994-7/2017 | H7N1 | PELPKGR↓GLF | G | Q | G |  | E | D |  | N |  | V |  | S |  | S |
| A/mallard/Kr/H673-4/2016 | H7N7 | PELPKGR↓GLF | G | Q | G |  | E | D |  | N |  | V |  | S |  | S |
| A/mallard/Kr/H840-2/2017 | H7N7 | PELPKGR↓GLF | G | Q | G |  | E | D |  | N |  | V |  | S |  | S |
| A/mandarin DK/Kr/H539-3/2016 | H7N7 | PELPKGR↓GLF | G | Q | G |  | E | D |  | N |  | V |  | S |  | S |
| A/mallard/Kr/H872-1/2017 | H7N7 | PELPKGR↓GLF | G | Q | G |  | E | D |  | N |  | V |  | S |  | S |
| A/green-winged teal/Kr/H873-5/2017 | H7N7 | PELPKGR↓GLF | G | Q | G |  | E | D |  | N |  | V |  | S |  | S |
| A/mandarin DK/Kr/H890-3/2017 | H7N7 | PELPKGR↓GLF | G | Q | G |  | E | D |  | N |  | V |  | S |  | S |
| A/mallard/Kr/H901-1/2017 | H7N7 | PELPKGR↓GLF | G | Q | G |  | E | D |  | N |  | V |  | S |  | S |
| A/spot-billed DK/Kr/H905-2/2017 | H7N7 | PELPKGR↓GLF | G | Q | G |  | E | D |  | N |  | V |  | S |  | S |
| A/mallard/Kr/H915/2017 | H7N7 | PESPKGR↓GLF | G | Q | G |  | E | D |  | N |  | V |  | S |  | S |
| A/spot-billed DK/Kr/H920/2017 | H7N7 | PELPKGR↓GLF | G | Q | G |  | E | D |  | N |  | V |  | S |  | S |
| A/common teal/Kr/H937-5/2017 | H7N7 | PELPKGR↓GLF | G | Q | G |  | E | D |  | N |  | V |  | S |  | S |
| A/spot-billed DK/Kr/H955-3/2017 | H7N7 | PELPKGR↓GLF | G | Q | G |  | E | D |  | N |  | V |  | S |  | S |
| A/mallard/Kr/WA245/2017 | H7N7 | PELPKGR↓GLF | G | Q | G |  | E | D |  | N |  | V |  | S |  | S |
| A/spot-billed DK/Kr/WA260/2017 | H7N7 | PELPKGR↓GLF | G | Q | G |  | E | D |  | N |  | V |  | S |  | S |
| A/mandarin DK/Kr/H987-3/2017 | H7N7 | PELPKGR↓GLF | G | Q | G |  | E | D |  | N |  | V |  | S |  | S |
| A/mallard/Kr/H836-10/2017 | H7N3 | PELPKGR↓GLF | G | Q | G |  | E | D |  | N |  | V |  | S |  | S |
| A/norther pintail/Kr/H895-3/2017 | H7N6 | PELPKGR↓GLF | G | Q | G |  | E | D |  | N |  | V |  | S |  | S |
| A/mallard/Kr/H906-3/2017 | H7N7 | PELPKGR↓GLF | G | Q | G |  | E | D |  | N |  | V |  | S |  | S |
| A/mallard/Kr/H1055-2/2017 | H7N7 | PELPKGR↓GLF | G | Q | G |  | E | D |  | N |  | V |  | S |  | S |
| A/mallard/Kr/H910/2017 | H7N7 | PELPKGR↓GLF | G | Q | G |  | E | D |  | N |  | V |  | S |  | S |
| A/mallard/Kr/H956-1/2017 | H7N7 | PELPKGR↓GLF | G | Q | G |  | E | D |  | N |  | V |  | S |  | S |
| A/mandarin DK/Kr/H993-10/2017 | H7N2 | PELPKGR↓GLF | G | Q | G |  | E | D |  | N |  | V |  | S |  | S |
| A/northern pintail/Kr/WB1833/2012 | H7N1 | PELPKGR↓GLF | G | Q | G |  | E | D |  | N |  | V |  | S |  | S |
| A/environment/Korea/A40-2/2012 | H7N1 | PELPKGR↓GLF | G | Q | G |  | E | D |  | N |  | V |  | S |  | S |
| A/environment/Korea/A01-1/2013 | H7N1 | PELPKGR↓GLF | G | Q | G |  | E | D |  | S |  | V |  | S |  | A |
| A/environment/Korea/A01-2/2013 | H7N7 | PELPKGR↓GLF | G | Q | G |  | E | D |  | N |  | V |  | S |  | S |
| A/environment/Korea/A36/2013 | H7N1 | PELPKGR↓GLF | G | Q | G |  | E | D |  | N |  | V |  | S |  | S |
| A/mallard/Kr/WB566/2013 | H7N7 | PELPKGR↓GLF | G | Q | G |  | E | D |  | N |  | V |  | S |  | S |
| A/environment/Kr/H432/2014 | H7N7 | PELPKGR↓GLF | G | Q | G |  | E | D |  | N |  | V |  | S |  | A |
| A/environment/Korea/H2254/2015 | H7N7 | PELPKER↓GLF | G | Q | G |  | E | D |  | N |  | V |  | S |  | S |
| A/environment/Korea/H2438/2015 | H7N7 | PELPKGR↓GLF | G | Q | G |  | E | D |  | N |  | V |  | S |  | S |
| A/mallard/Kr/A15/2016 | H7N7 | PELPKGK↓GLF | G | Q | G |  | E | D |  | N |  | V |  | S |  | S |
| A/spot-billed DK/Kr/AQ09/2016 | H7N7 | PELPKGR↓GLF | G | Q | G |  | E | D |  | N |  | V |  | S |  | S |

^a^H7 numbering was used. HA, hemagglutinin; PB, polymerase basic; M, matrix; NS, nonstructural; Kr, Korea; DK, duck.

Supplementary table 3. Real-time RT-PCR results for oropharyngeal and cloacal shedding

| Isolate | Group | Sample | Virus shedding (Ct value)^a^ | | | | |
| --- | --- | --- | --- | --- | --- | --- | --- |
|  |  |  | 3 dpi | 5 dpi | 7 dpi | 10 dpi | 14 dpi |
| A/mandarin Dk/Kr/H539-3/2016 (H7N7) | Challenged | OP | 2/5  (35.7, 32.0) | 3/5  (38.0, 32.9, 33.1) | 1/5  (38.81) | 0/4 | 0/4 |
|  |  | CL | 3/5  (28.1, 37.0, 26.6) | 4/5  (28.8, 30.4, 28.9, 31.2) | 4/5  (30.3, 30.6, 29.9, 33.1) | 2/4  (36.2, 35.4) | 0/4 |
|  | Direct contact | OP | 1/3  (34.4) | 2/3  (39.2, 27.3) | 0/3 | 0/3 | 0/4 |
|  |  | CL | 0/3 | 1/3  (30.2) | 2/3  (34.6, 26.2) | 2/3  (30.4, 33.3) | 0/4 |
| A/mallard/Kr/H982-6/2017 (H7N7) | Challenged | OP | 0/5 | 0/5 | 0/5 | 0/5 | 0/5 |
|  |  | CL | 0/5 | 0/5 | 0/5 | 0/5 | 0/5 |
|  | Direct contact | OP | 0/3 | 0/3 | 0/3 | 0/3 | 0/3 |
|  |  | CL | 0/3 | 0/3 | 0/3 | 0/3 | 0/3 |

Values shown are (number of infected birds)/(number of inoculated birds) in the challenge group, and (number of infected birds)/(number of naïve birds) in the direct-contact group. Values in parentheses are Ct value. A sample was considered positive for IAV if the Ct value was less than 40. Ct value of negative samples was not shown. The inoculation dose for chickens was 10^6^ EID_50_/0.1 ml. CL, cloacal; dpi, days post-infection; Dk, duck; Kr, Korea, OP, oropharyngeal.

^a^Ct value, cycle threshold.
